# Between two stools: preclinical research, reproducibility, and statistical design of experiments

**DOI:** 10.1186/s13104-022-05965-w

**Published:** 2022-02-21

**Authors:** Penny S. Reynolds

**Affiliations:** Department of Anesthesiology, College of Medicine, Department of Small Animal Clinical Sciences, College of Veterinary Medicine, University of Florida, Gainesville, FL 32610 USA

**Keywords:** Reproducibility, Experimental design, Statistics, Animal-based research

## Abstract

Translation of animal-based preclinical research is hampered by poor validity and reproducibility issues. Unfortunately, preclinical research has ‘fallen between the stools’ of competing study design traditions. Preclinical studies are often characterised by small sample sizes, large variability, and ‘problem’ data. Although Fisher-type designs with randomisation and blocking are appropriate and have been vigorously promoted, structured statistically-based designs are almost unknown. Traditional analysis methods are commonly misapplied, and basic terminology and principles of inference testing misinterpreted. Problems are compounded by the lack of adequate statistical training for researchers, and failure of statistical educators to account for the unique demands of preclinical research. The solution is a return to the basics: statistical education tailored to non-statistician investigators, with clear communication of statistical concepts, and curricula that address design and data issues specific to preclinical research. Statistics curricula should focus on statistics as process: data sampling and study design before analysis and inference. Properly-designed and analysed experiments are a matter of ethics as much as procedure. Shifting the focus of statistical education from rote hypothesis testing to sound methodology will reduce the numbers of animals wasted in noninformative experiments and increase overall scientific quality and value of published research.

## Introduction

“…I think we’re falling between two stools at the moment.… I think we have to take a step backward and address the basics of our game.”

––Donal Lenihan 25 Nov 2020, RTÉ Rugby Podcast, on Ireland’s need to revise training strategy following a string of defeats to England.

Criticism of much animal-based preclinical research has centred on reproducibility issues and poor translation [1, 2]. Causes are systemic and multifactorial, and include poor model fidelity, clinical irrelevance of target biomarkers or molecular pathways, and between-lab disparities in models and procedures [3, 4]. Difficulties in verifying and replicating methodology [5] and methodological issues related to poor statistical design and analysis are also major contributors [6–10]. Translational failure has massive economic repercussions. Advances in therapeutic agents or diagnostics development are more than offset by multimillion-dollar losses in investment, and ultimately unsustainable research and development costs [6, 11, 12]. There is also a significant ethical component to these failures. If questionable methodology produces biased or invalid results, evidence derived from animal-based research cannot be a reliable bridge to human clinical trials [13]. It is difficult to justify the continued use of millions of animals each year if the majority are wasted in non-informative experiments that fail to produce tangible benefit.

In this commentary, I suggest that preclinical research has ‘fallen between two stools’, by not conforming to either clinical trial or agricultural research traditions or skillset camps, and with little of the rigour of either. The solution is a return to the basics for statistical educators and consultants: statistical training explicitly tailored to non-statistician investigators, and coverage of statistical issues and topics relevant to preclinical research. In particular, I urge a change in focus from statistics as ‘just maths’ to statistics as process. I argue that reform of introductory statistics curricula along these lines could go far to reverse statistical pathologies common to much of the preclinical research literature.

## Main text

### Two stools of competing traditions

The clinical trial and agricultural/industrial research traditions show considerable divergence in focus and methodology. *Clinical trials* are performed when there is uncertainty regarding relative efficacy of a specific clinical intervention. They are constrained by the necessity to minimize subject risk of mortality and severe adverse events. In general, clinical trials tend to be relatively large and simple, with only two or a few comparator interventions randomly assigned to many subjects, ideally representative of the target population. Although clinical trials have a history going back several hundred years (e.g. [14]), the randomized controlled trial (RCT) as the gold standard was a relatively recent development, with the first modern RCT performed in 1946 [15, 16], and formalisation only in the late 1970s. Lagging implementation was due in part to resistance to the so-called “numerical approach” by supporters of the non-randomised “let's-try-it-and-see” attitude to clinical research problems [17, 18]. Meanwhile, methodology for observational studies was being developed in parallel. Cohort studies in particular have had a key role in epidemiological investigations of carcinogenic and environmental hazards when RCTs are not feasible [19]. Because factors are not randomly assigned to subjects*,* inferring causality requires stringent methodological safeguards for minimising confounding and bias [15, 20, 21].

In contrast, *agricultural/industrial designs* are characterised by small sample sizes and multiple factors studied simultaneously. In addition to randomisation, key design features include replication and blocking (‘local control’), coupled with formal statistically-structured arrangements of input variables, such as randomized complete block and factorial designs [22]. Agricultural designs were developed primarily by Sir Ronald Fisher in the early half of the twentieth century. These principles were subsequently extended to industrial experimentation by George Box and collaborators [23]. Industrial experiments are further distinguished by *sequential implementation* (data from a small or restricted group of runs in the original experiment can be used to inform the next experiment), with prompt feedback (*immediacy*), allowing iteration and relatively rapid convergence to target solutions [24]. For these applications, variable screening and model building are both of interest, and ‘design’ is essentially the imposition of a statistical model as a useful approximation to the response of interest [23, 25].

### Preclinical studies: between the stools

Animal-based research studies are unique for the explicit ethical obligation to minimise the numbers of animals used. Application of Three Rs (Replacement, Reduction, Refinement) principles are based on the premise that maximum scientific value should be obtained with minimal harms [26]. However, over-emphasis on numbers reduction has contributed to underpowered experiments generating unreliable, and ultimately noninformative, results [27, 28].

Small sample sizes, large variability, multi-group comparisons, and the exploratory nature of much preclinical research suggest that study designs should be more aligned with the agricultural/industrial tradition. Fisher-type designs (such as randomised complete blocks and factorials) are suitable for purpose and have been vigorously promoted [12, 29–33], as have procedural methods for controlling variation without increasing sample size [34], and design features that increase validity [1, 35]. However, these methods seem to be virtually unknown in the preclinical literature [7, 8, 36–38]. Two-group comparisons more typical of clinical trials are common, although unsuited to assessing multiple factors with interactions. Informal examination of introductory textbooks and statistics course syllabi suggest that knowledge gaps are due in part to sparse formal training in experimental design, and neglect of analytical methods more suited to preclinical data. Compounding these problems is lack of general statistical oversight. Unlike human-based studies [39], few animal research oversight committees in the USA have access to properly qualified biostatisticians, statistical analysis plans and study preregistration are not required, and protocol review criteria vary considerably between institutions [40].

### Statistical pathologies in the preclinical literature

Bad statistical practices are very deeply entrenched in the preclinical literature. Many of the major errors observed in the research literature involve statistical basics [41–43]. Statistics service courses tend to emphasise mathematical aspects of probability and null hypothesis significance testing at the expense of non-mathematical components of statistical process [44–46]. Consequently, it is now part of the belief system of many investigators that ‘statistical significance (P < 0.05)’ is the major criterion for assessing biological importance of results, and that P-values are an intrinsic property of the biological event or group of animals being studied [47]. As a result, there is over-reliance on rote hypothesis testing and P-values to interpret results. Related pathologies include reporting of orphan inexact P-values with no context, P-hacking, N-hacking, selective reporting, and spin [41, 48].

A second problem area is poor understanding by investigators of basic statistical concepts and operational definitions. Statistical terms are frequently conflated with lay meanings, confused with other technical definitions, or ignored. Concepts that seem especially misunderstood include ‘study design’, ‘randomisation’, ‘cohort’, ‘unit of analysis’, and ‘replication’. To investigators, ‘study design’ refers primarily to descriptions of technical methodology and materials, e.g. [49]. To applied statisticians, ‘study design’ is the formal arrangement and structuring of independent or predictor variables hypothesized to affect the response or outcome of interest. A good study design maximizes the experimental signal by accounting for diverse sources of variability [31, 50, 51]), and incorporates specific design features to ensure results are reliable and valid, such as correct specification of the unit of analysis, relevant outcome measures, inclusion and exclusion criteria, and bias minimization methods [8, 35, 52]. ‘Randomisation’ to statisticians is a formal probabilistic process that minimizes selection bias and effect of latent confounders, and is the cornerstone for statistical inference. In contrast, randomisation in preclinical studies seems to be frequently misinterpreted in the lay sense of ‘unplanned’ or ‘haphazard’ [53], or is likely not performed at all [8, 38, 54, 55]. The common habit of referring to a group of animals subjected to a given treatment or intervention as a ‘cohort’ likely reflects non-random allocation of subjects to a defined intervention group, an invalid and confounded assignment strategy [56]. The term ‘cohort’ actually refers to groups of subjects in observational studies, where group membership is defined by some common characteristic [19]. It does not refer to experimental treatment groups with group allocation determined by randomisation. The meaning of ‘unit of analysis’ is virtually unknown, or confused with biological and observational units [56–58]. ‘Replication’ is frequently interpreted solely as duplication of the total sample size for ‘reproducibility’ [59], rather than as an independent repeat run of each combination of treatment factors [25].

A third area of concern is that the conventional statistical arsenal of t-tests, ANOVA, and χ^2^ tests [60, 61] is unsuited for analysing ‘problem’ data typical of many preclinical studies. ‘Problem’ data include non-gaussian, correlated (clustered, nested, time dependencies), or non-linear data; data that are missing at random or due to dropout or attrition; data characterised by over-representation of true zeros; and high-dimensional data. A major deficiency that must be addressed is the focus of introductory courses on methods virtually unchanged since the 1950s, with little coverage of modern methods more appropriate for such data [8, 35, 44].

Finally, there is little attention paid to methods for identifying diverse sources of variation during experiment planning. Research papers rarely report auxiliary variables and conditions related to animal signalment, environment, and procedures only indirectly related to the main experiments, e.g. [62]. Such variables contribute to unpredictable effects on animals and experimental results, resulting in uncontrolled variation that obscures true treatment effects. For example, systematic investigations of factors contributing to survival time in mouse models of amyotrophic lateral sclerosis suggested that claims for therapeutic efficacy were most likely due to the effects of uncontrolled variation rather than actual drug effects [12, 29, 33].

## Outlook

Lack of knowledge on the part of investigators is related to training deficiencies on the part of statistics educators. The solution is a return to the basics: statistical education that meets the needs of non-statistician investigators, and curricula addressing design and data issues specific to preclinical research. This is hardly new: in 1954, John Tukey identified as essential that “statistical methods should be tailored to the real needs of the user” [63], and this has been repeated in the decades since [9, 44, 46, 64, 65]. Investigators still identify better training in statistics and statistical methods as a high priority [9, 64]. The June 2021 report by the Advisory Committee to the Director of the National Institutes of Health (NIH-ACD) made five major recommendations to improve rigor and reproducibility of animal-based research, among which was recognition of the need for “modern and innovative statistics curricula relevant to animal researchers” [9].

What do researchers need? The poor internal validity characterising much preclinical research [66] reflects poor understanding of the upstream basics of statistically-based study design and data sampling strategies. Unreliable downstream results cannot be rescued by fancy analyses after the fact, as Fisher himself warned [67]. Therefore, the concept that good statistical principles must be built in during planning and before data are collected must be introduced and reinforced. This can be accomplished first, by more appropriate training of entry-level researchers with courses and topic coverage more attuned to specific need, and second by removal of longstanding barriers (such as cost and academic credit) to early consultation with appropriately-training statisticians. Early formal involvement of applied statisticians in the planning process must be encouraged and rewarded [9, 68].

Statistical educators and consultants must be re-educated to better address actual research needs. ‘Statistics’ is neither just maths nor an analytical frill tacked on to a study after data have been collected. Instead, statisticians must structure instructional materials to reflect the basic tenets of statistical process: design before inference, and data quality before analysis [69]. Data curation skills are also part of good statistical practice [46], identified as such for nearly a century [70]. These practices are not strongly mathematical, and unfortunately statisticians tend to be uninterested in non-mathematical procedures [46, 71]. Second, service courses must shift away from pedagogical approaches common to applied maths or algebra, where uncritical analysis of a data set leads to a fixed ‘correct’ solution [46, 71, 72]. Procedural change could be accelerated by statisticians becoming more aware of best-practice expectations though evidence-based planning [73] and reporting [74] guidelines. These tools can direct early-stage study planning to ensure that procedures strengthening study validity can be incorporated [4, 35, 74, 75].

Properly designed and analysed experiments are an ethical issue [28, 66, 69]. Shifting the focus of statistical education from rote hypothesis testing to sound methodology should ultimately reduce the numbers of animals wasted in noninformative experiments and increase overall scientific quality and value of published research.

## Data Availability

Not applicable.

## Abbreviations

3Rs: Replacement, Reduction, Refinement
NIH-ACD: Advisory Committee to the Director of the National Institutes of Health
RCT: Randomised controlled trial
